# Analysis of user trends in digital health communities using big data mining

**DOI:** 10.1371/journal.pone.0290803

**Published:** 2024-08-26

**Authors:** Ron Keinan, Efraim Margalit, Dan Bouhnik

**Affiliations:** Department of Computer Science, Lev Academic Center, Jerusalem College of Technology, Jerusalem, Israel; CCET: Chandigarh College of Engineering and Technology, INDIA

## Abstract

Camoni, the largest digital health community in Israel, involves thousands of patients in the decision-making process concerning their illness and treatment. This approach reflects the recent global shift towards digital tools that combine professional information with social networking capabilities to enable problem-solving, emotional support, and knowledge sharing. Digital health communities serve as an invaluable resource for individuals seeking to learn more about their health, connect with others with shared experiences, and receive encouragement. Our research investigates user trends in digital health communities using the Camoni platform as a case study. To this end, we compile a comprehensive database of 12 years of site activity and conduct a large-scale analysis to identify and assess significant trends in user behavior. We observe several significant trends concerning different genders engagement and note a narrowing of gaps between men and women users’ participation and publication volume. Furthermore, we find that younger users have become increasingly active on the platform over time. We also uncover unique gender-specific behavior patterns that we attempt to characterize and explain. Our findings suggest that the rise of digital health communities has accelerated in recent years, reflecting the public’s growing preference to take a more active role in their medical care.

## Introduction and background

### Introduction to digital health communities

The Internet has brought about several far-reaching changes affecting our daily lives. It has drastically changed how people communicate with others. Social media platforms such as Facebook, X, and Instagram have become popular channels for people to stay in touch with friends and family, share thoughts and opinions, and even form new relationships [1, 2]. These new platforms have had a profound impact on the way we exchange information, engage with others, and consume entertainment and media [3]. The rise of digital tools has enabled patients to access a wealth of medical information and connect with others dealing with similar health issues. Digital health communities have emerged that provide patients with knowledge and support to manage their health more independently [4], provide and receive support, and share information [5]. These networks enable members to share their experiences, communicate with others facing similar health problems, and benefit from mutual support and insights [6].

As a result, patients and their families have become increasingly active participants in their medical treatment. With the help of information provided by medical professionals or online resources, patients are now a vital part of the decision-making process regarding their illness and treatment [4]. The traditional model that saw medical experts as the exclusive owners and suppliers of medical knowledge has been challenged by the emergence of patients as active contributors to their healthcare.

The advent of digital tools and the accompanying rise of use in digital health communities have transformed the way patients participate in their healthcare. Patients have become a significant source of medical information in their own right, and online social networks have become a crucial source of support and information for those dealing with health issues.

This research centers on the examination of digital health communities, aiming to delineate user behavior by associating it with their characteristics. Our underlying hypothesis posits a direct correlation between users’ characteristics and the extent of their engagement, manifested through their writing and overall activity within digital health communities.

More general public platforms are also used to generate information regarding public health. For example, Facebook deals in many fields, including health, and studies [7, 8] have proven that a lot of medical information can be compiled with the help of the platform. Some also used unique Facebook abilities such as analyzing "Like" comments for collecting health data [9]. Reddit also used research based on text analysis to extract information from the discussions on the network to identify trends in public health [10–12]. However, it is important to note that this is done more in dedicated groups as well as customized applications on Facebook such as health surveys and questionnaires.

This study focuses on "Camoni", an Israeli website that consists of digital health communities.

### Camoni–An Israeli platform for digital health communities

In this exploratory research, we analyze the social interactions that occur on Camoni, an Israeli online social network (OSN) of health communities.

Camoni is a platform that provides free access to knowledge regarding health issues for patients, families, and caregivers. It is the largest platform for digital health communities in Israel (Camoni website, 2023), comprising 41 distinct communities each focused on specific medical conditions such as diabetes, cancer, and obesity. These communities are managed by experts and community leaders, who curate content that includes detailed guides, news and research updates, patients’ rights, personal blogs, and discussion groups. The site was founded to empower patients and their families to take an active role in managing their medical conditions [13]. Every community has active administrators who are professionals in the relevant field, and they rigorously review and delete unreliable or irrelevant information to uphold the quality of content shared on the platform [14].

In Camoni users can ask questions, receive answers, and share their experiences about various diseases and medical fields. Each community contains thousands of posts, where the post usually represents a question/problem raised by a user. For each post, there can be comments that usually express an emotional or professional response to the post, as well as comments from the post writer who responds to the answers and adds additional questions.

Our study focuses on some of the demographic characteristics of the users in the digital health communities in Camoni. We examined the behavior of users and the distribution of posts along the timeline, while identifying different patterns that may indicate social trends. To the best of our knowledge, this is the first analysis of a Hebrew-language digital health community that examines posts and comments in connection with these issues.

Camoni is a project of The Gartner Institute, a community interest company free from commercial interests. The institute is part of the Sheba Medical Center in Ramat-Gan, which is the biggest medical center in Israel.

The institute’s mission is to assist the health system and the Israeli Ministry of Health in designing informed healthcare policy [14, 15]. Unlike other medical sites, Camoni’s content focuses on patients and not medical staff, whose role is only to supervise and advise [16].

To gain insight into the social interactions taking place within Camoni over time, we conducted a scraping of the site’s data–posts, comments, and user details. Our study covers an extended timeframe, from the establishment of Camoni in 2010 until the end of 2021, which allows us to examine trends and changes in the online health community in Israel as it evolved.

### User differences in digital health communities

Social networks can serve as a bulwark of psychological and social support [17] in times of crisis such as COVID-19, and they have become a major source of medical information worldwide [18]. It is to be expected that different users will make different use of such platforms, based on their needs, and we attempted to identify such patterns on the "Camoni" website as other researchers have identified on similar platforms. For example, different genders have been found to exhibit different patterns of behavior in digital health communities. Liu [19] discovered that the content produced and consumed by each gender varied, as well as the role users assumed on the platform. According to Klemm [20], gender differences in online health communication may be linked to the topics discussed. Their investigation of three cancer support groups (breast cancer, prostate cancer, and a third mixed-gender group) revealed that women tend to offer support and encouragement, while men tend to share biomedical information. More recent research has also revealed a correlation between gender and information needs and concerns in digital health communities [21].

In general, men and women differ in their behavior on online platforms. Putzke [22] studied male and female users on the popular music sharing and social media platform Last.FM, finding that they differ both in terms of speed of adoption and patterns of usage. Roy [23] showed that Indian male and female users use the Internet to satisfy different gratification and use needs, such as self-development, user-friendliness, wide exposure, and relaxation. Another study found that men were more likely to want to be a part of an online travel community as opposed to women [24]. A big research study in the USA showed that men are much more active on the Internet specifically when expressing their opinions [25]. Others [26, 27] proved that women demonstrate a higher degree of digital engagement compared to men, especially in topics associated with mental health issues such as depression and anxiety. A recent analysis proved major differences in the usage of social media depending on gender, age, and demographic characteristics [28].

Moreover, men and women have been found to have different approaches with regard to healthcare. Goodwin [29] claims that women’s central roles within the family enable them to become health managers or promoters of overall family health. Narang and Singhal [30] observe that men in India tend not to be involved in reproductive or antenatal healthcare. Recent research has identified gender disparities in terms of social support and mental resilience [31]. They showed that male students have higher levels of emotional intelligence than female students, but that female students report a greater need for social support during the pandemic than male students, a fact that caused more symptoms of depression and anxiety among women.

Age is another important factor affecting Internet usage and participation in digital health communities. This was supported by survey results demonstrating a positive correlation between younger age and increased average daily Internet usage [32]. Additionally, research has shown a significant association between age and the digital divide, revealing that older adults in Lisbon are less likely to engage with technology due to functional and attitudinal barriers, notwithstanding the influence of age and education on the adoption of mobile phones and computers [33]. This disparity may be attributed to the diverse barriers faced by older computer users, which can be classified into intrapersonal, interpersonal, structural, and functional constraints. These barriers vary across different age segments, thereby affecting their interaction with computer-mediated information technology [34].

On the other hand, at times this is not so obvious. For example, in a study analyzing posts in a digital health community for those with a specific autoimmune liver disease, Lasker [35] discovered that, contrary to their expectations, age was unrelated to either frequency of posts or the content of the messages. Another research highlights that while younger users are more likely to engage in activities like instant messaging, online gaming, and social media, email usage is nearly universal across all age groups. This suggests that internet use can vary depending on the specific activity [36].

Different generations are characterized by starkly different approaches in how they interact with technology. In this study, we will attempt to identify trends related to the generation of users. As our guide, we will use the following overview found in [37–39]:

- iGen/Generation Z: Born in 1996 and after, they are the first true digital natives who grew up with technology and social media. They are diverse, entrepreneurial, and socially conscious.
- Millennials/Generation Y: Born from 1977 to 1995, they are often characterized as confident, collaborative, and creative. They came of age during a time of rapid technological advancement and are the most educated generation yet.
- Generation X: Born 1965 to 1976, they are independent, adaptable, and skeptical of institutions. They were the first generation to grow up in dual-income households and often experienced a more hands-off parenting style.
- Baby Boomers: Born between 1946 to 1964, they are often characterized as optimistic, idealistic, and hardworking. They grew up during a time of significant social and cultural changes, such as the Civil Rights Movement and the rise of feminism. They are currently in or entering retirement age.

Big data mining can be used to extract meaningful insights, identify trends, and detect user behavior patterns over time.

### Big data mining

The term big data refers to datasets that are too large or complex to be easily processed using traditional data processing methods. These datasets may come from a wide range of sources, including social media, and may include unstructured data, such as text, images, and video, which can be particularly challenging to work with [40].

Big data mining refers to the process of extracting useful insights and knowledge from large and complex datasets. This is typically done using advanced statistical and machine learning techniques, as well as specialized software tools designed to handle extremely large datasets [41] Big data mining is also popular in research regarding health/medical data [42–44]. One of the key benefits of big data mining is the ability to identify patterns and trends in large and complex datasets that may not be immediately apparent using traditional data analysis methods.

However, big data mining also presents several challenges [45]. For example, the sheer volume of data can make it difficult to store, process, and analyze efficiently. The variety of data sources and formats can also make it challenging to integrate and analyze data effectively. Additionally, new concerns about data privacy and security as well as the potential for bias and errors in data analysis can arise.

### The research goals

Our research is designed to accomplish two primary goals. First, we aimed to create a comprehensive database of all the information available on the Camoni site, which would facilitate an analysis of the quantity and content of the data. Second, we conducted an extensive investigation of the data using big data analysis tools, with the primary objective of identifying significant trends on the site, particularly in terms of demographic differences.

The findings from our study not only shed light on the needs of patients dealing with diseases but also highlight the strengths and weaknesses of the digital health community. This information can aid in providing better access to information for patients and improve the overall patient experience. Moreover, our research provides a basis for future studies to delve further into the subject and uncover new insights.

## Methodology

### The research question

Is there a discernible relationship between user characteristics on the "Camoni" website and their activity, as reflected in the frequency of posts and comments on the timeline, as well as the community’s growth over the first 12 years of its activity? Additionally, how do demographic factors such as gender, age, occupation (medical staff vs. non-medical users), and user tenure(time since joining the website) influence user behavior within the digital health community?

### The research hypotheses

#### The main research hypothesis

H1: Characteristics of users on the "Camoni" website, such as gender, age, and user status, have a direct effect on their activity on the website. Specifically, certain subgroups exhibit increased activity in terms of joining the website and contributing posts.Sub-hypotheses:

H2: Since its inception in 2010, the Camoni website has experienced a significant increase in public activity, which whill be reflected in higher levels of user engagement, including a rise in the frequency of posts and comments related to health topics, as well as a growth in the overall number of users joining the community over time.H3: Senior users will display average levels of activity on the site due to their familiarity with diseases and treatment methods. An increase of activity will be found among the younger users as well, owing to their technological orientation.

H4: Gender differences will not significantly impact user activity on the Camoni website, as cultural norms and digital habits within the Israeli population appear to be uniform across genders.

H5: We expect that initially, upon establishment, medical staff members will be found to be the most active participants on the site, given its establishment by the Gartner Institute, a division of the Sheba Medical Center. We expect however to find that, over time, the participation of the general population, including patients and their families, increased vastly.

### The examined population

Camoni hosts a large and diverse set of digital health communities that have remained active over many years, making it an excellent source of data to examine for long-term trends. Unlike other medical websites that focus on specific areas such as geriatrics or oncology, Camoni covers a wide range of fields and diseases in the medical realm.

There are 2 types of forums on the site—communities and groups. Communities are opened by the administrators of the site who closely monitor them. Groups can be opened on any individual topic by different users and are more loosely supervised.

We examined all the 41 professionally managed health communities on the Camoni website, whose topics include cancer, eating disorders, blood pressure, diabetes, heart disease, depression, and more. Our sample consisted of all the content posted on the website between 2010–2021, comprising over 400,000 posts and comments contributed by more than 25,000 users.

The extensive scope of our research enabled us to obtain a comprehensive understanding across various medical domains over a substantial 12-year timeframe associated with "Camoni." The breadth of our investigation contributes to its objectivity, ensuring that it accurately captures trends without succumbing to biases influenced by specific professional fields. Furthermore, the oversight and management of the 41 communities by site administrators attest to a supervised and elevated professional standard, thereby enhancing the credibility and quality of our findings.

Although the website hosts another type of forum, hundreds of user-created groups on various topics, we chose to focus on formal digital communities since they are better organized and more specifically targeted toward medical issues.

To facilitate more general research and trend identification, we grouped the 41 communities into 6 domains based on related medical fields. These domains were developed in collaboration with a medical team and represent major medical disciplines. The domains chosen are Oncology, Chronic Diseases, Dermatology, Orthopedics and Joints, Internal Medicine, and Mental Health Therapy.

### Data creation and analysis

Processing of the data was divided into three main stages: scraping the data from the website, parsing the data and building databases by topic, and processing the data to identify trends and changes.

#### Scraping camoni contents

To access the data from the Camoni website, we faced the challenge of working without a structured API for all content. This required a careful study of the website’s URL structure to identify templates for each section. In total, we identified approximately 600,000 potential URLs.

Using custom code, we sent an HTTP request to each URL and saved the content to an HTML file. However, during the advanced stages of processing, we realized that some of the data was missing from the saved files. After further investigation, we determined that the data was being pulled from another source using JavaScript commands. To obtain the complete data, we re-downloaded all information using the Selenium Hidden Chrome Driver infrastructure to generate data as viewed in the browser.

To avoid triggering the website’s server defenses, which could detect our requests as a potential DDOS cyber-attack [46], we implemented a wait time of about 1.5 seconds between each request.

While many of the potential URLs were empty or returned incomplete data, we ultimately obtained approximately 130,000 data binaries, comprising 97,000 post and comment files, and 32,000 user data files.

#### Data preparation

Our next step involved processing the obtained data. To accomplish this, we utilized the Python package Beautiful Soup, which enabled us to parse HTML files [47]. Our goal was to extract key data from each page of a post, comment, and user details. This involved matching HTML tags to the data viewed in the browser and developing an algorithm to extract the necessary data and store it in a uniform and organized database (DB).

We used the ’pandas’ [48] and ’xlwt’ [49] packages in Python to save the DB as Excel files. The DB included a central table that contained posts, their comments, and accompanying metadata, such as the community in which they were written, the author, timestamps, and more. Additionally, a second table included user data, such as gender, date of joining the site, communities of which they were a member, and user status (medical staff member, patient’s family member, or coping with the disease themselves).

#### Data analysis

After constructing the database, we proceeded to analyze the collected data, with a focus on identifying trends and shifts in user behavior. Our analysis mainly focused on the characteristics of the website’s users: gender, age, and seniority (activity time on the website).

We considered two primary aspects of user behavior: we focused on the number of posts, the length of posts, and the time since joining the community as indicators of user activity and interaction. These metrics provide insights into the level of engagement and participation of users within the digital health community. The quantity metric signifies quantitative activity, while length is associated with depth, reflecting factors such as the amount of medical information conveyed or the level of empathy expressed. Our decision to analyze these variables was formed by the need to comprehensively assess user behavior and its impact on the dynamics of the Camoni platform.

To perform this effort, we employed the use of Tableau, a powerful business intelligence tool designed for data visualization and analysis [50]. We entered the raw data into Tableau and utilized its diverse functionalities to conduct the entire analysis. Specifically, we utilized Tableau to extract metrics such as word count, post count, and various timeline tracking data. Additionally, Tableau facilitated the identification of relationships between different user characteristics, such as gender and age, and the amount and content of posts they authored.

Additionally, Tableau also allowed us to highlight different indicators such as activity trends over time, gender-based variations in activity levels, and several other data representations that provided valuable insights into our analysis. It is used in many data analysis-heavy research, including those dealing with health data [51].

In our statistical analysis, we employed both Cramer’s V test and the chi-square test to examine the relationships and associations within our data. The Cramer’s V test is a measure of association used to determine the strength of the relationship between two categorical variables. It allows us to assess the degree of correlation between variables, providing valuable insights into patterns and dependencies within the data. Additionally, the chi-square test is a statistical method used to determine whether there is a significant association between two categorical variables. By comparing observed and expected frequencies, the chi-square test enables us to assess the independence of variables and identify any significant deviations from expected values[52].

#### Data source

Camoni functions as a publicly accessible website where users are permitted to publish posts and comments. These contributions are viewable to anyone on the Internet, regardless of their registration status on the platform. The terms of use explicitly allow for non-commercial utilization of the content. Additionally, the site administrators have outlined their commitment to ensuring the appropriateness of published content. They diligently review posts and remove any publications deemed inappropriate, containing inaccurate medical recommendations, or featuring content associated with violence or pornography, among other criteria.

Following these site policies, our engagement with Camoni is contingent upon utilizing content that users have expressly agreed to share and that has undergone approval by the site administration. Our utilization adheres strictly to privacy restrictions and other stipulations outlined in the platform’s terms of use.

Given the publicly accessible and anonymized nature of the data obtained from the "Camoni" platform, ethical approval was not required for this study. However, our research adhered strictly to all relevant regulations and guidelines outlined by "Camoni", ensuring compliance with ethical standards.

## Findings

### A generic camoni dataset

The primary objective was to construct a comprehensive database that would facilitate not only the present investigation but also future inquiries and subsequent research on digital health communities in Israel. This objective was effectively accomplished: we succeeded in creating a database structured in a tabular form, encompassing all the relevant information on the website from 2010 to 2021, such as posts, comments, associated metadata, and user profiles that include comprehensive details. The dataset consists of 4 central tables:

The user table contains over 23,000 user records, including characteristics—gender, age, medical status, date of joining the site, and more.

The table of communities, contains 41 records of the name of the community, the name of the medical domain to which it belongs, and the number of users who are members of the community.

The posts table contains all the posts published independently on the site, and a total of over 64 thousand records. For each post, the date of publication, the author’s name, title and text separately, its length in words and its length in characters, the community in which it was published, the number of comments on the post, and finally all the comments that were published concerning that post.

The comments table, which contains all the posts and comments published on the site in general, each as an independent paragraph of text, and in total this table has over 386 thousand records. The table broadly reflects the writing activity on the site, without differentiation between the initiating author and the responding author.

### Users and usage trends in camoni

Several areas were analyzed to uncover intriguing trends or patterns. The most notable and illuminating trends and changes were linked to variations in activity in the digital health communities based on demographic characteristics such as gender, age, user status, and seniority of authors, especially over time.

### Posts vs. comments separated by gender/domain and year

**Fig 1:**
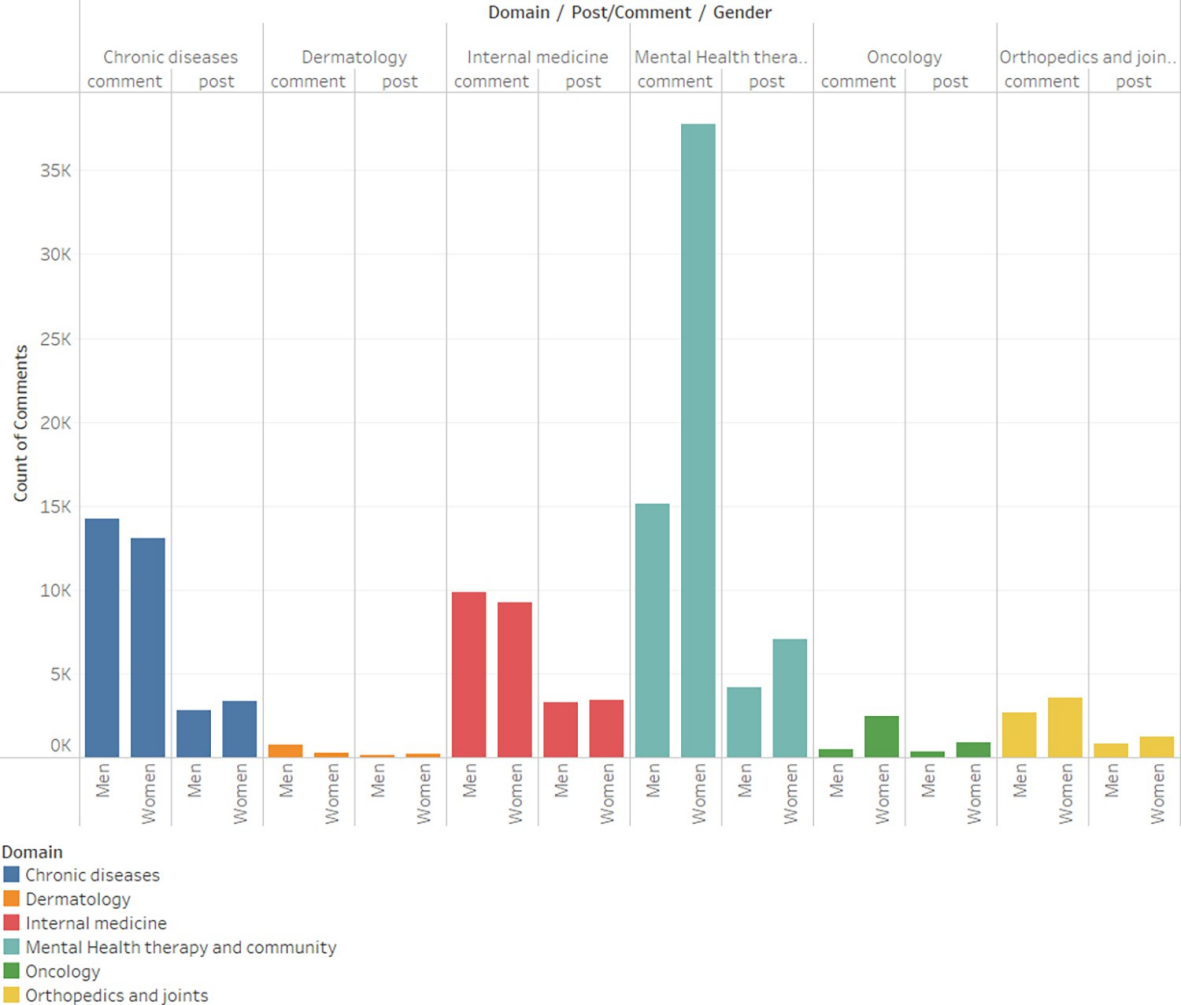
Posts comments count by gender and domain.

Fig 1. Presents the gender-based breakdown of the number of posts versus comments across various domains. It reveals that, in general, users tend to write more comments than posts, a trend that is particularly pronounced for women. Furthermore, the mental health domain exhibits the largest discrepancy in this regard.

In order to examine the hypothesized relationship between gender and Post/Comment, a chi-square test for independence was executed. The statistical outcomes demonstrate a significant association between the two variables (*x*^2^_(1)_ = 55.65, *p*<0.001, *Cramer*′*s V* = 0.02) with the observed relationship characterized by a Cramer’s V coefficient of 0.02, suggesting a small but discernible effect size.

Examination of the Mental Health therapy and community domain reveals a relationship that is slightly more pronounced, yet still characterized by a weak effect size (*x*^2^_(1)_ = 338.99, *Cramer*′*s V* = 0.07).

**Fig 2:**
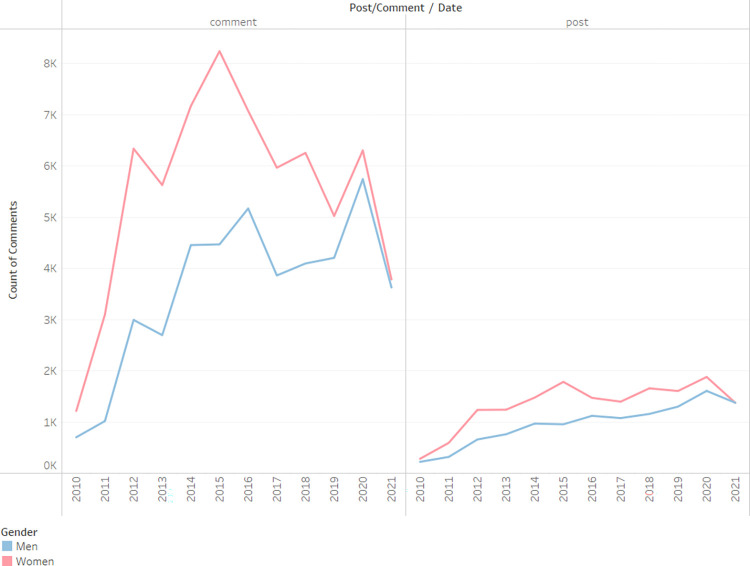
Posts comments count by gender and year.

It is interesting to note the timeline of publication distribution between posts and comments for men and women. For all years, women lead in both the number of comments and posts respectively, as seen in Fig 2. Over time, however, men have significantly narrowed this gap, with the number of comments and posts now being on par with that of women.

### Men focus on the mental health domain

**Fig 3:**
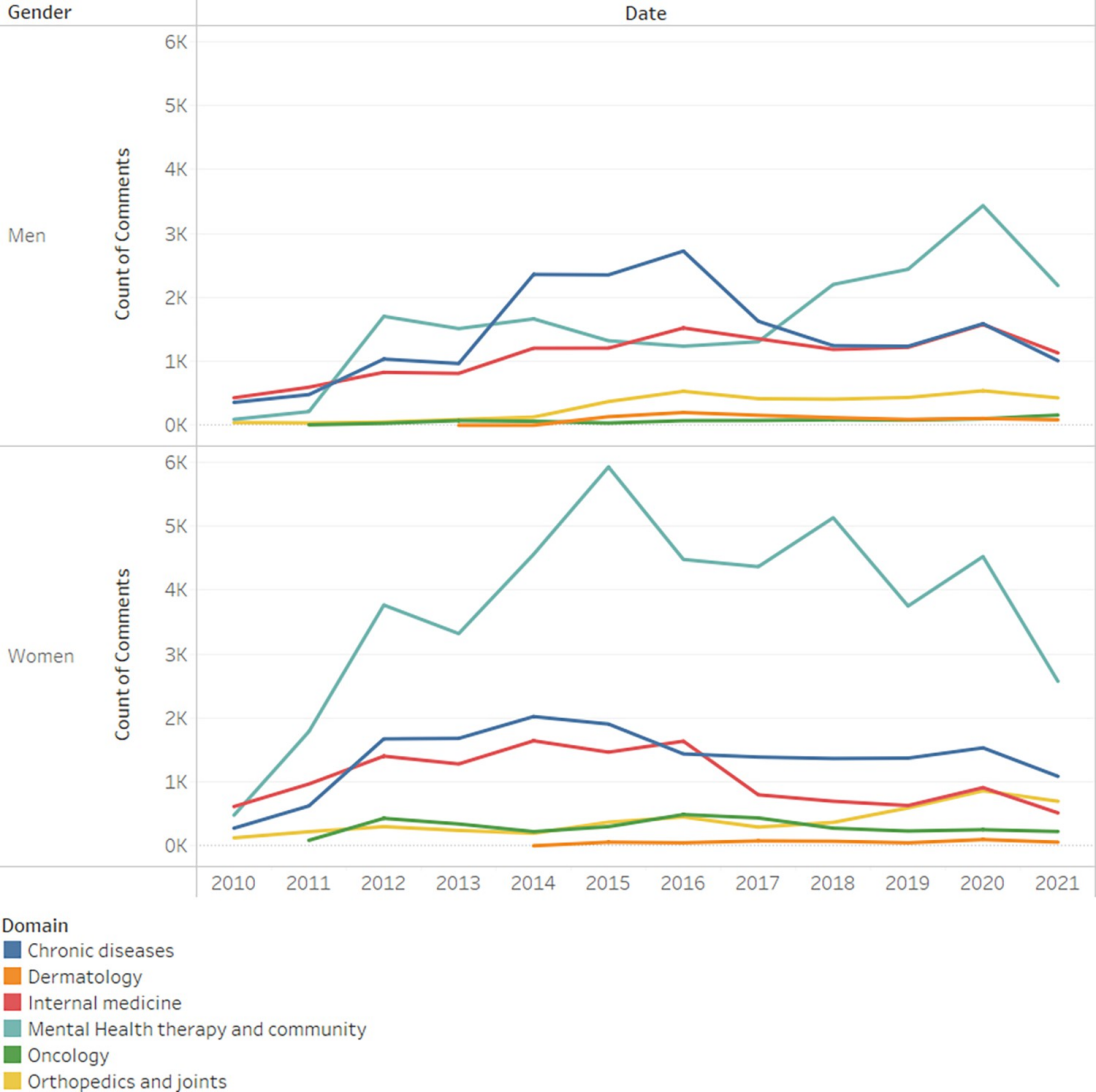
Count of comments by year and domain.

The number of comments broken down by domain, is presented in Fig 3 and reveals the most active domain to be Mental Health Therapy and Community. When examining differences across genders, we find that while women were always most active in mental support communities, men were more active in Chronic Diseases (such as Diabetes and Sclerosis Multiple), until that domain was overtaken in 2017 by Mental Health.

In order to investigate the hypothesized relationship between gender and domain, a chi-square test for independence was conducted. The statistical outcomes indicate a substantial association between the two variables (*x*^2^_(1)_ = 6639.7982, *p*<0.001, *Cramer*′*s V* = 0.22). The magnitude of the Cramer’s V coefficient suggests a moderate effect size, emphasizing the strength of the observed relationship.

### Gender differences in the mental health domain

**Fig 4:**
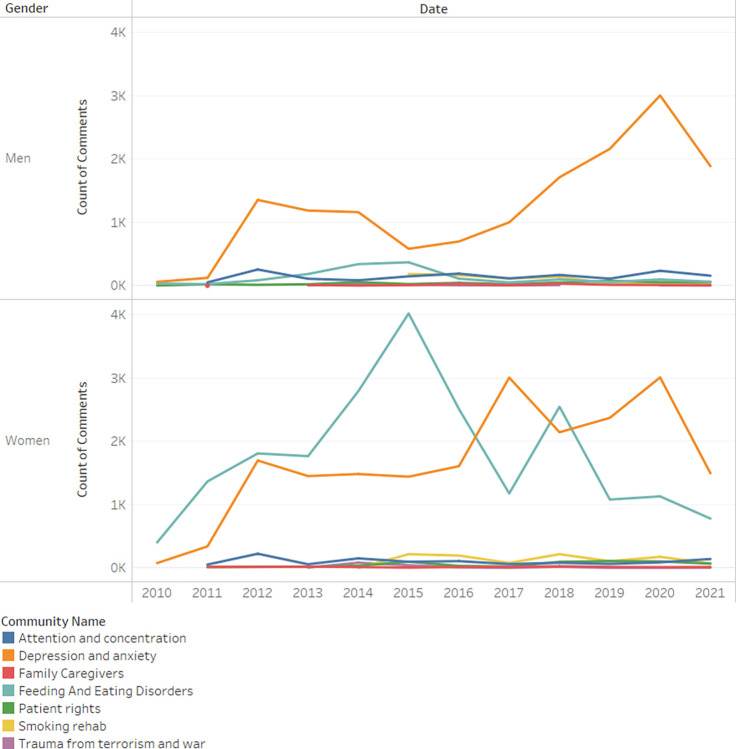
Count of comments mental domain by year and domain.

Fig 4 illustrates the Mental Health Therapy and Community domain, divided into seven distinct communities, and breaks down the number of comments posted within each by year. It is noteworthy that the depression community is the most active community among men, while the eating disorder community is generally the most active among women.

In order to investigate the hypothesized relationship between gender and community, a chi-square test for independence was conducted. The statistical outcomes indicate a substantial association between the two variables (*x*^2^_(1)_ = 9825.82, *p*<0.001, *Cramer*′*s V* = 0.39). The magnitude of the Cramer’s V coefficient suggests a moderate effect size, emphasizing the strength of the observed relationship.

### Gender differences in post-length

**Fig 5:**
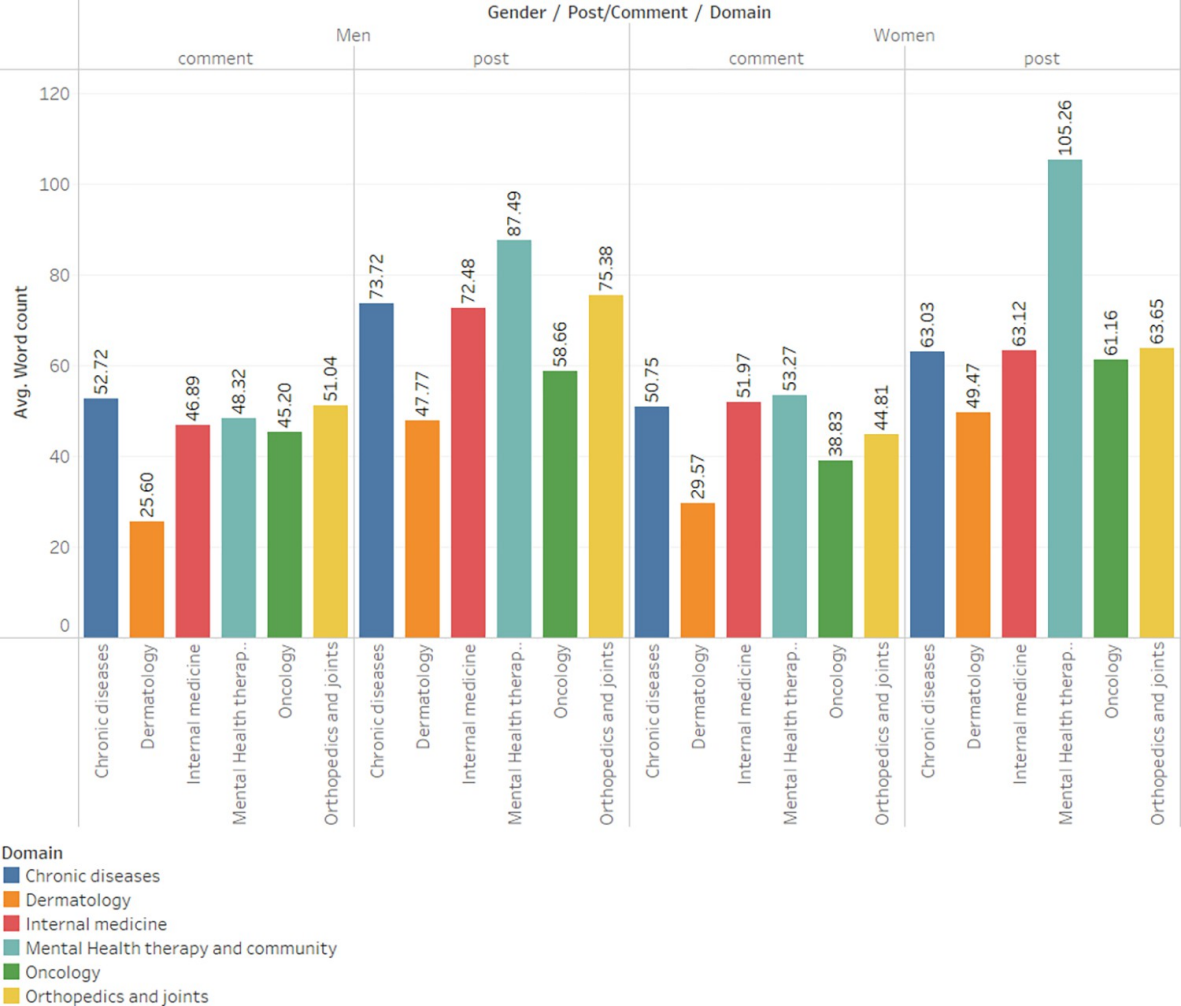
Average word count by gender and domain.

**Fig 6:**
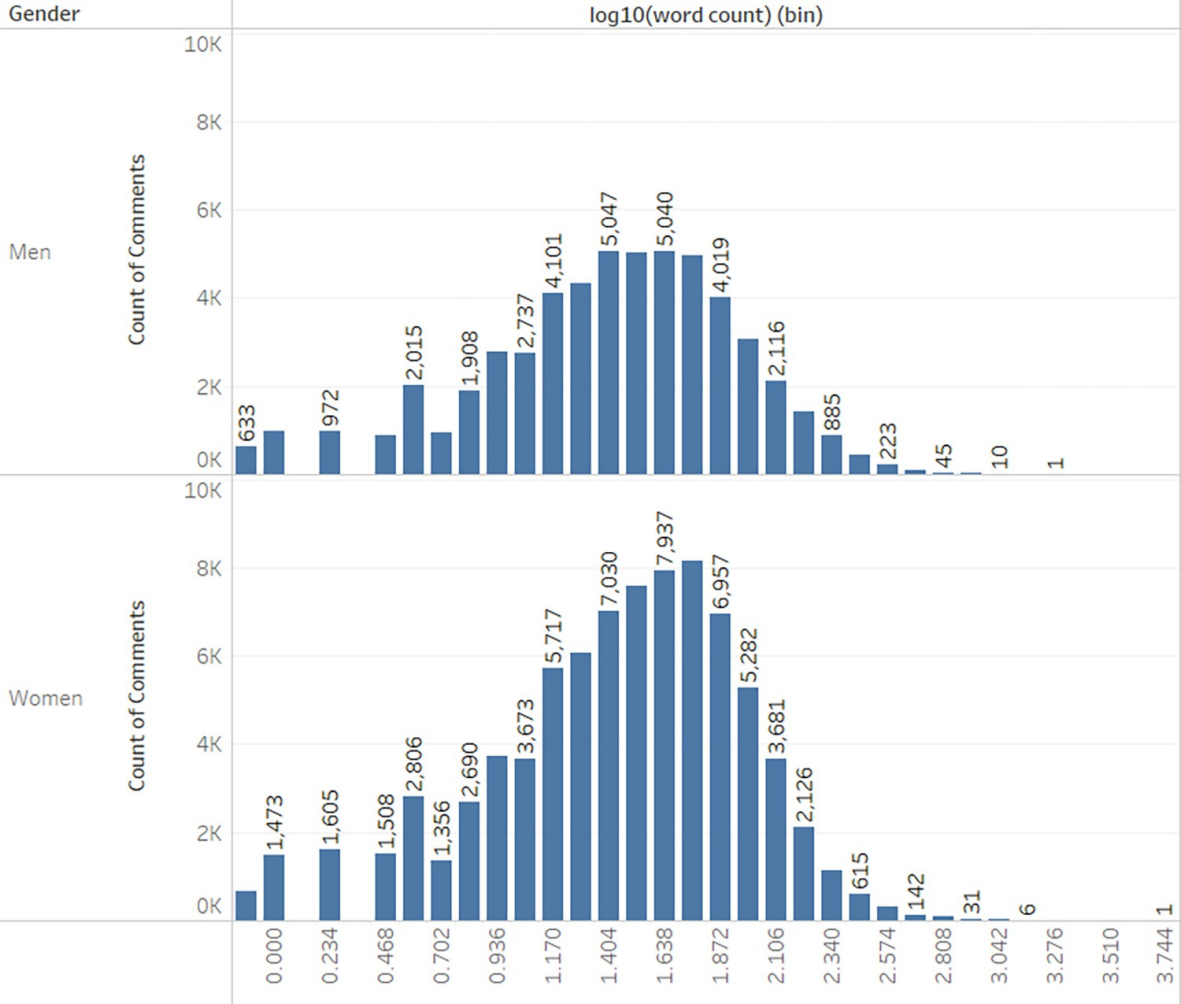
Average word count by gender and domain with log10.

Analyzing the average length of posts and comments based on gender and domain reveals an intriguing pattern in Fig 5. Women tend to write longer posts in the mental health field (Average of 135.8 words for men and 158.5 for women), while men typically produce lengthier content in clinical medical domains such as internal medicine or oncology (Average of 126.4 words for men and 113.8 for women),

By applying logarithmic base 10 transformations to normalize the length of the posts, it becomes apparent that the distribution of lengths tends towards a Gaussian or normal distribution, as seen in Fig 6. We adopted the method recommended by Raban & Rabin [53].

### The average age of users

**Fig 7:**
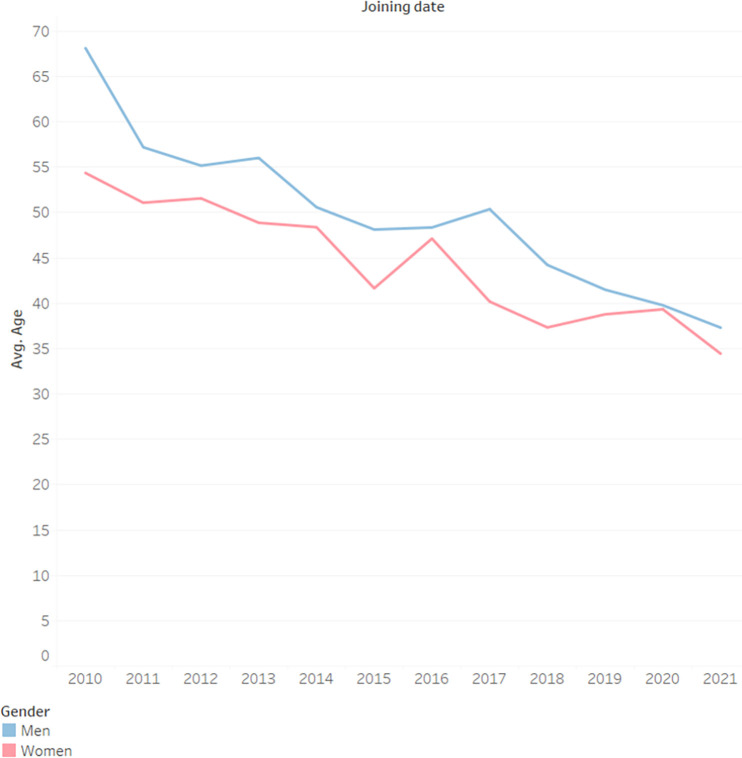
Average age by seniority and gender.

In analyzing the average age of users who contribute posts and comments each year, we observe a consistent trend of declining age, indicating a greater interest in health-related topics on the part of younger generations. Fig 7 shows the average age of users by their joining year (seniority on the site).

**Fig 8:**
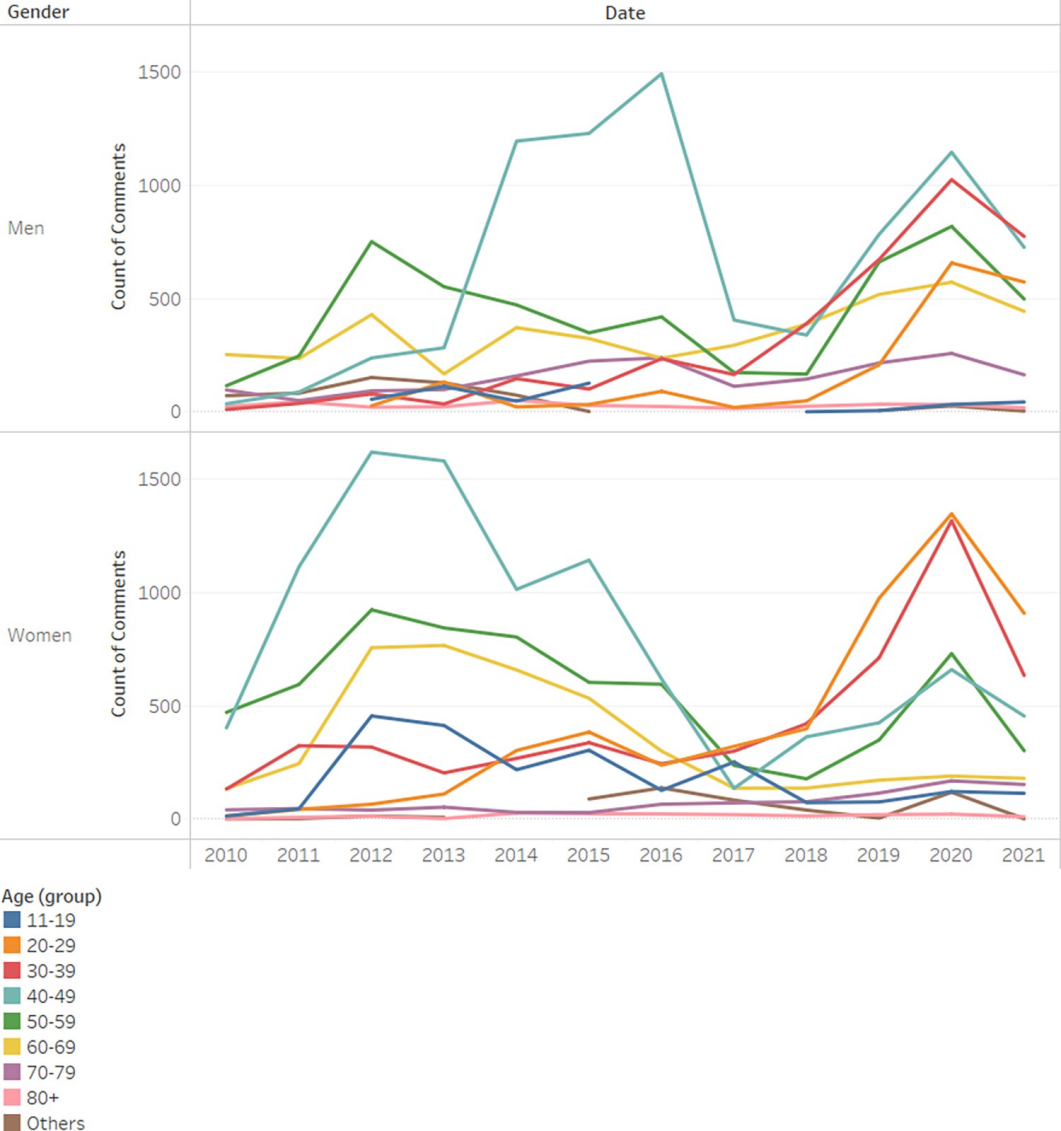
Count of comments by age and year.

Divided into age ranges and not just average age, in Fig 8 we witness a transition for the most active female users from ages 40–60 to ages 20–40. For men, the change is more moderate: the most active male users in recent years are 40–50. In the main, women are active at younger ages than men.

In order to investigate the hypothesized relationship between gender and age group, a chi-square test for independence was conducted. The statistical outcomes indicate a substantial association between the two variables (*x*^2^_(1)_ = 2587.99, *p*<0.001, *Cramer*′*s V* = 0.21). The magnitude of the Cramer’s V coefficient suggests a moderate effect size, emphasizing the strength of the observed relationship.

**Fig 9:**
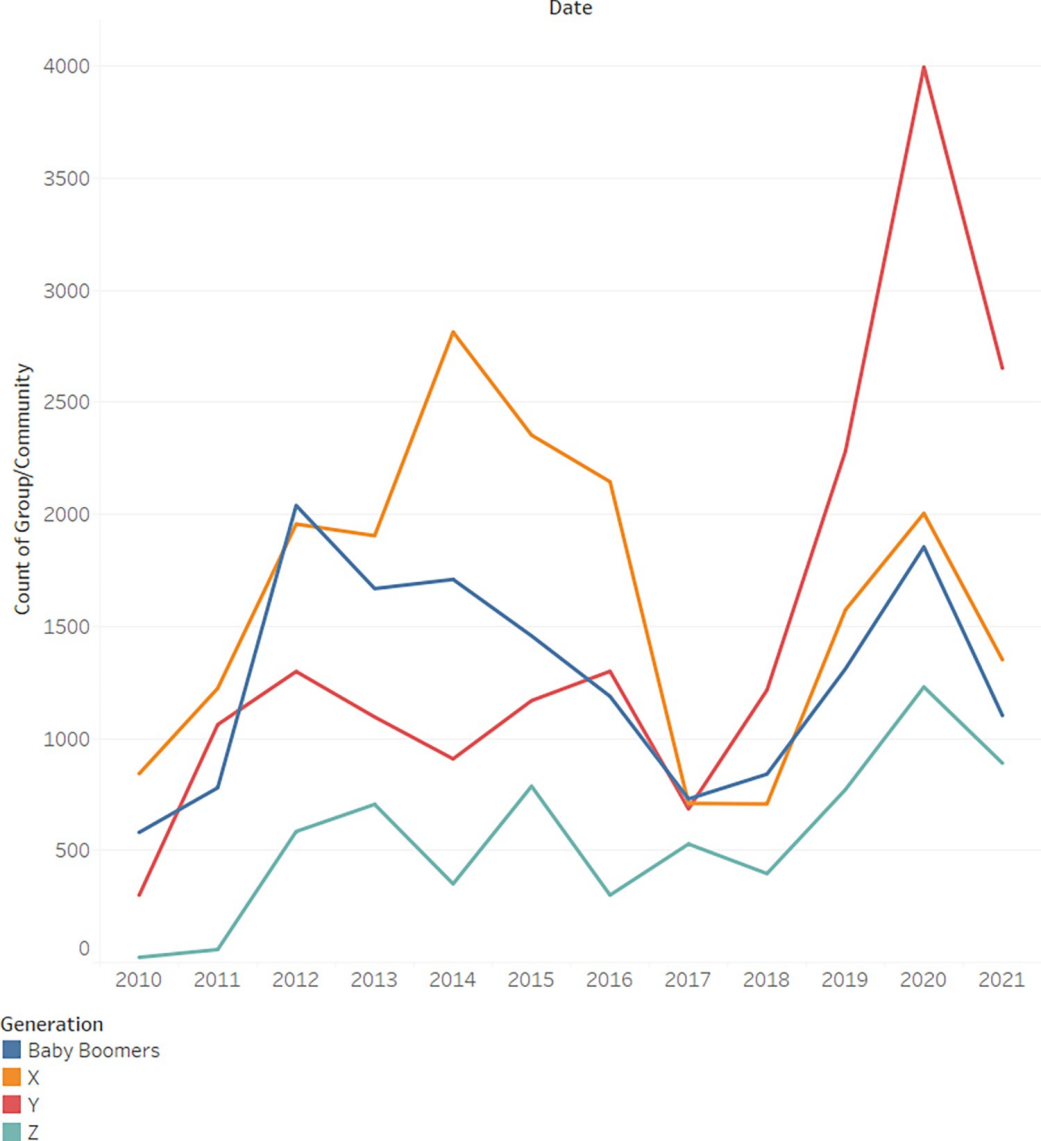
Count of comments by user generation.

**Fig 10:**
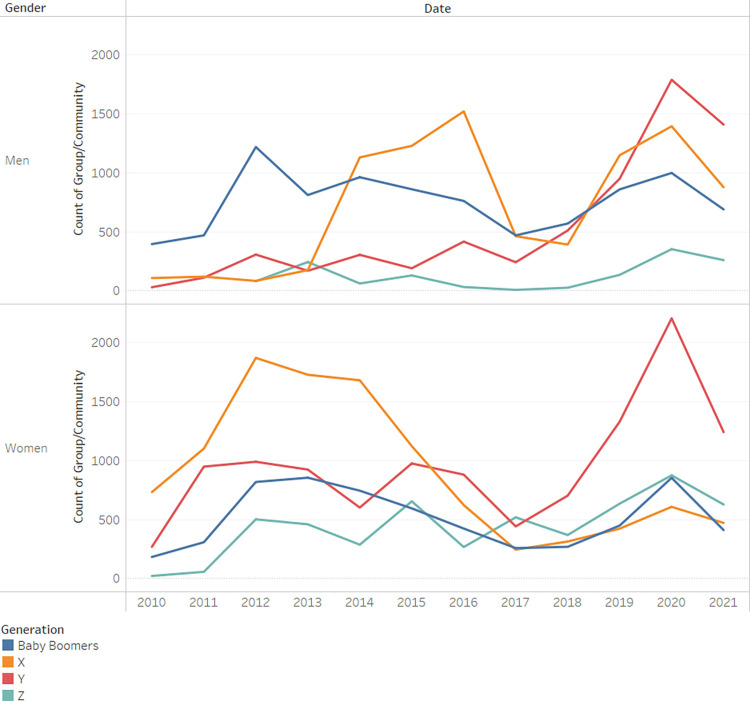
Count of comments by user generation and gender.

When we examine(Fig 9) the distribution of active users grouped according to their generation, the Y generation is seen to grow more and more active and in recent years even replace the X generation as the most active group of users. When broken down by gender(Fig 10), it seems that for women this phenomenon is even more pronounced.

In order to investigate the hypothesized relationship between gender and generation, a chi-square test for independence was conducted. The statistical outcomes indicate a substantial association between the two variables (*x*^2^_(1)_ = 3485.33, *p*<0.001, *Cramer*′*s V* = 0.24). The magnitude of the Cramer’s V coefficient suggests a moderate effect size, emphasizing the strength of the observed relationship.

### Effect of seniority on post average length

**Fig 11:**
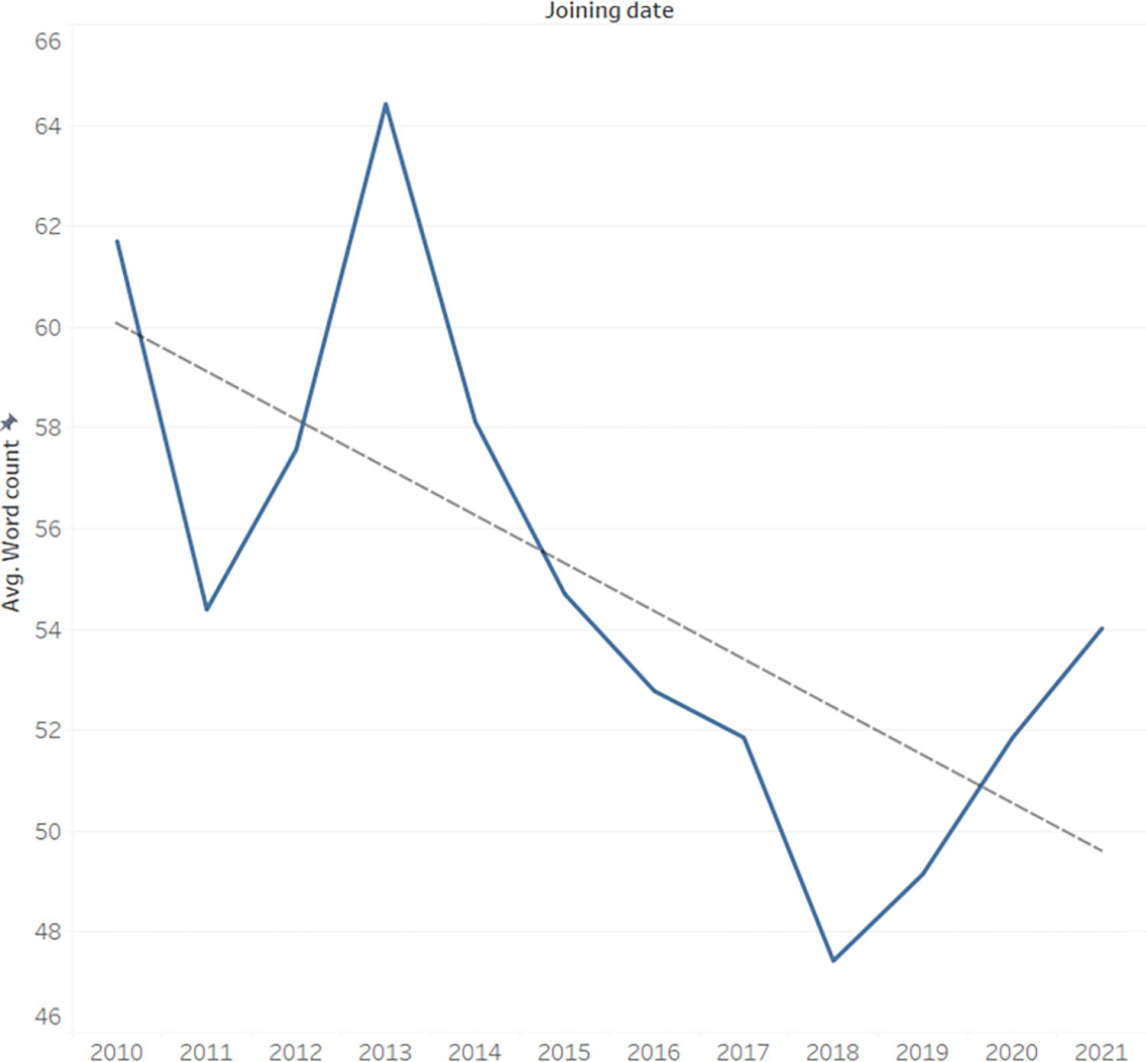
Average word count by seniority.

When analyzing the average post length based on when users joined the site, it appears(Fig 11) that there is a general declining trend in the length of posts for those who joined more recently, up to 2018. However, after 2018 this trend reverses itself, and for writers joining in 2018 or later, there is a noticeable uptick in the average length of their posts the more recently they joined.

### User status and gender/post length

Users are divided into 3 status types: users dealing with the disease themselves, family members of a patient, or medical staff members.

Looking at the distribution of status between men and women in Table 1, patients themselves are seen to be the leaders of both genders. However, women comprise a greater percentage of active family members on the site compared to men.

**Table 1:** User count by user status.

Gender	Status	Absolute	Relative %
**Women**	Medical staff	133	4.69
	Family member of a patient	489	17.27
	Dealing with the disease	2,208	78.02
	Total	2,830	100
**Men**	Medical staff	112	5.67
	Family member of a patient	200	10.12
	Dealing with the disease	1,663	84.2
	Total	1,975	100
**Total**		**4805**	**100**

In order to examine the hypothesized relationship between gender and status, a chi-square test for independence was executed. The statistical outcomes demonstrate a significant association between the two variables (*x*^2^_(1)_ = 49.17, *p*<0.001, *Cramer*′*s V* = 0.10) with the observed relationship characterized by a Cramer’s V coefficient of 0.10, suggesting a small but discernible effect size.

When examining (Table 2) the number of comments written by users by gender and status, it can be seen that the total number of comments is nearly identical for men and women. However, female medical staff are more than twice as active as the male medical staff. Male patients, in turn, are more prolific than their female counterparts.

**Table 2:** Comments count by user status and gender.

Gender	Status	Count of Comments	Relative %
**Women**	Dealing with the disease	13,215	69.33
	Family member of a patient	1,310	6.87
	Medical staff	4,535	23.79
	Total	19,060	100
**Men**	Dealing with the disease	15,563	81.58
	Family member of a patient	1,489	7.80
	Medical staff	2,023	10.60
	Total	19,075	100
**Total**		**38,135**	**100**

In order to examine the hypothesized relationship between gender and Post/Comment, a chi-square test for independence was executed. The statistical outcomes demonstrate a significant association between the two variables (*x*^2^_(1)_ = 1165.22, *p*<0.001, *Cramer*′*s V* = 0.17) with the observed relationship characterized by a Cramer’s V coefficient of 0.02, suggesting a small but discernible effect size.

Concerning the average length of a post, Table 3 shows that female medical staff write longer posts than their male counterparts, who write the shortest posts compared to the other types of users, with a difference of about 10 words on average between the genders. Male family members tend to write longer posts compared to female family members, whose posts are on average 11 words shorter than the men.

**Table 3:** Average word count by user status and gender.

Status / Gender post word number	Women	Men	AVG
Dealing with the disease	50.79	47.53	49.16
Family member of a patient	46.88	56	51.44
Medical staff	55.38	44.81	50.09
**AVG**	51.01	49.44	50.22

## Discussion

Our primary hypothesis, H1, appears substantiated, suggesting the feasibility of pinpointing demographic characteristics that exhibit heightened activity relative to others. This observation is discernible in both the user count and the quantity of posts authored. A significant portion of the results substantiated the proposed hypothesis, with a moderate effect size observed, ranging from a Cramer’s V coefficient of 0.21 to 0.38.

As expected by H2, Camoni’s user activity has grown steadily over 12 years. This is in line with the general increase in interest in digital health communities and health documented in the literature [4–6, 18, 19]. Such a phenomenon could also reflect the intensification of digital activity on the Internet and particularly in health-related subjects following the COVID-19 epidemic in 2020–2021, which raised awareness about health, treatment, and prevention. Bierman’s research [18] did demonstrate the need for social media connections during the pandemic.

Contrary to our hypothesis H4, we found several significant differences in how men and women used the website, although these differences generally became smaller over time. Across all users–comments are more frequent than posts, especially in the domain of mental health. This trend is especially true for women, suggesting that they tend to seek and offer emotional support more than men as found in Klemm [20]. Women not only provide more social support than men but also require more social support themselves. These aspects are not contradictory but rather represent different facets of social interaction, encompassing both giving and receiving support. However, we found that the gender gap is decreasing, and men are now responding nearly as much as women. This observation is corroborated by the minimal effect size, registering at 0.01 across all domains and slightly higher at 0.07 specifically within the Mental Health domain.

Both genders in recent years were also most active in the same domain: Mental Health. Although this was always the most active domain for women, it only became so for men in 2017. This is contrary to some research we examined that found men as more active internet users [25] and correlates to later research [26] that showed women are more active users compared to men, in particular in areas related to mental health such as depression and anxiety [27].

Within the Mental Health domain, the depression and anxiety community saw an especially high number of posts/comments for both genders. The authors in the eating disorder community, however, were mostly women, perhaps because thin body ideals are more common for women in Western society. Our study seems to stand in contrast to the findings of Barros and Fontenla [31] that men exhibit greater mental resilience. Women tended to write longer posts and comments, especially in the area of mental health. This could reflect a greater degree of social support for those struggling with illness, as found in Barros and Fontenla [31]. Furthermore, we found that a much higher percentage of the female users were family members (17.3% of women compared to 10.1% of men). This finding would seem to reinforce the proposition that women, with their unique caregiving responsibilities and experience, can take a more active role in supporting and managing healthcare for other members of their family [29].

The reason female medical staff wrote twice as many comments as male (4,532 compared to 2,023) medical staff is unclear. Similarly, it is not clear why women’s longest posts are from medical staff and men’s longest posts are from family members.

We did not find evidence to support our hypothesis H5 that over time more users would be patients themselves and not medical staff, as the medical staff were never the principal contributors on the site. Throughout all the years of the site’s activity, those dealing with the disease were the most active users.

Regarding H3, we found that the average age of users on Camoni was found to decrease over time, strengthening the conclusions of several researches [32–35] that individuals use the Internet more the younger they are, and in fact, suggesting that this trend has intensified in recent years. We believe that this is due to the reason that patients seem to be taking advantage of the new tools available to them to take a more active role in their medical care, especially younger patients. Women in particular are involved at an even earlier age than young men. Thus our hypothesis that younger users would become more active in recent years was affirmed.

Generation Y may be more active than Generation X in digital health communities due to their greater comfort with technology, increased health awareness, and the accessibility of digital platforms. Additionally, Generation Y’s use of social media and potential differences in age-related health concerns could also contribute to their greater activity in these communitiesWomen exhibit greater activity than men across all generations, except for "Baby Boomers," where men contribute more posts than women. The Cramer’s V coefficient of 0.24 indicates a moderate association in this context. The reason is unclear.

Users who joined the site at an earlier point tended to write longer posts, possibly because they have more experience in understanding and addressing the needs of the community and are thus able to author more comprehensive and complete comments. As such, we accurately predicted that more experienced users would be more active on the site.

It seems that compared to other platforms, such as Facebook [7–9], or Reddit [10–12] in the communities of "Camoni" there is a simple and extensive engagement in health issues. There is no need to compile surveys, address users, or count likes to receive information. Moreover, text analysis remains essential despite the absence of diverse subjects within these networks, as their exclusive focus on health issues warrants a comprehensive understanding of user interactions. Given that the primary objective of the website is the public dissemination of health-related concerns, the increasing utilization of this platform by the Israeli public signifies a growing demand for a tailored response to their health needs, distinguishing it from other prevalent networks in Israel.

## Summary and future work

Digital health communities have emerged as an essential resource for patients and their families seeking medical information and support. The growing trend of patients relying on alternative sources of information can enhance the treatment process and empower individuals to take a proactive role in managing their own and their family’s health. Israel in particular has experienced substantial growth in activity in digital health communities like Camoni. In order to best inform and treat patients, healthcare systems and policymakers can use the insights gained regarding how the different genders and age groups interact with these new tools to improve health outcomes. It is important to note that this behavior may be due to various factors beyond a proactive attitude towards personal health, such as dissatisfaction with medical treatment or a desire for more information due to perceived deficiencies in the provision of health services. This point should be examined in depth in further research.

This study represents a pioneering effort using extensive data mining techniques to analyze the behavior of digital health communities in Israel and can serve as a foundation for future studies on this database or others.

In this manuscript, we have presented the foundational components of our research, encompassing the establishment of the dataset and a comprehensive analysis of site trends. As we proceed with the research, we aim to delve into additional phenomena, including investigating the dynamics between writers and commenters, exploring the impact of a user’s status on the communities to which they belong, and addressing other pertinent aspects. Furthermore, it is important to note that our study does not capture the inclusion of passive users and their behaviors could offer valuable insights into usage patterns within digital health communities. Moreover, we intend to incorporate an in-depth analysis of the content itself, employing machine learning algorithms for classification. This additional layer of analysis aims to fortify or challenge the conclusions drawn from the study, relying not only on metadata but also on a nuanced examination of the textual content.

While much research highlights the potentially negative effects of increased media consumption, it is encouraging to discover the positive impact these social networks can have as well. Individuals are increasingly comfortable sharing their personal struggles with others and offering support through online social networks. This is especially beneficial for those grappling with anxiety and mental health issues, who may hesitate to seek help face-to-face. It is worth examining the potential for adverse consequences, which may also emerge.

Finally, this study reflects the extensive activity that exists in social networks for health-related purposes. Such activity conveys optimism and hope, suggesting an increasing trend of individuals taking charge of their health and fostering a sense of solidarity through the sharing of knowledge and support for those facing similar medical challenges.
